# Treatment of Osseous Defects after Mandibular Third Molar Removal with a Resorbable Alloplastic Grafting Material: A Case Series with 1- to 2-Year Follow-Up

**DOI:** 10.3390/ma13204688

**Published:** 2020-10-21

**Authors:** Minas Leventis, Efstathia Tsetsenekou, Demos Kalyvas

**Affiliations:** Department of Oral and Maxillofacial Surgery, Dental School, National and Kapodistrian University of Athens, 2 Thivon Street, Goudi, 115 27 Athens, Greece; tsetsenekou2004@yahoo.com (E.T.); demkal@dent.uoa.gr (D.K.)

**Keywords:** mandibular third molars, bone regeneration, β-tricalcium phosphate, calcium sulfate, bone gain

## Abstract

Mandibular third molar (M3) surgical extraction may cause periodontal complications on the distal aspect of the root of the adjacent mandibular second molar (M2). Patients older than 26 years with periodontal pathology on the distal surface of the M2 and a horizontal/mesioangular impacted M3 may benefit from bone regenerative therapy at the time of surgery. In this prospective case series, an alloplastic fully resorbable bone grafting material, consisting of beta-tricalcium phosphate (β-TCP) and calcium sulfate (CS), was used for the treatment of the osseous defects after the removal of horizontal or mesioangular M3s in 4 patients older than 26 years. On presentation, the main radiological finding in all patients, indicating periodontal pathology, was the absence of bone between the crown of the M3 and the distal surface of the root of the M2. To evaluate the treatment outcome, bone gain (BG) was assessed by recording the amount of bone defect (BD) at the time of surgical removal (T0) and at the time of final follow-up (T1) 1 or 2 years post-operatively. The healing in all cases was uneventful, with no complications associated with the use of the alloplastic grafting material. Clinical and radiological examination at T1 revealed that all extraction sites were adequately restored, with significant BG of 6.07 ± 0.28 mm. No residual pathological pockets on the distal surface of the M2 were detected. Pocket depth (PD) at T1 was 2 ± 0.71 mm. Within the limitations of this case series, the results suggest that β-TCP/CS can support new bone formation at M3 post-extraction sites where bone regeneration methods are indicated, thus reducing the risk of having persistent or developing new periodontal problems at the adjacent M2.

## 1. Introduction

The presence of third molars is common in humans, and M3s are frequently found to be fully or semi- impacted in the bone [1]. The presence of a M3 that failed to erupt or is partially erupted may cause a plethora of problems, and indications for M3s removal include acute or chronic infection, pain, caries, prevention or repair of periodontal defects, pathology associated with cystic degeneration and/or neoplastic transformation of the dental follicle, and facilitation of orthodontic treatment [2,3]. The literature also suggests that even for asymptomatic and disease-free impacted or semi-impacted M3s in young adults, there is an accumulative high risk for extraction in the future [4]. As a result, the surgical removal of M3s is one of the most commonly performed procedures in oral surgery, and more than 10 million third molars are extracted every year in the United States [3].

Although after the extraction of a M3 the socket is generally healed spontaneously by formation of new bone, several clinical parameters may affect the level of bony healing, and surgical removal of third molars has been associated with the risk of having persistent or developing new periodontal defects on the distal aspect of the adjacent M2 [5]. One of the most important factors seems to be the age of the patient at the time of M3 removal and younger patients (age < 25) have a higher probability of uneventful healing [2,3].

Moreover, the positioning of a partially or fully impacted M3, as well as the periodontal status distally to the root of the M2 and the presence of bone between the M2 and the M3 are important parameters. A partially impacted M3 exposed to the oral environment is more prone to chronic infection, which may cause severe periodontal attachment loss on the distal aspect of the M2 [2]. Similarly, deeply impacted M3s in direct contact to the adjacent molar may lead to incomplete osseous healing and periodontal defects after their surgical extraction. It has been shown that in patients older than 26 years with a mesioangular or horizontal impaction and pre-existing periodontal defects, the periodontal healing of the M2 may be complicated resulting to intraosseous defects and deep periodontal pockets after M3 removal. According to the literature the majority of M3s indicated for surgical removal are mesially or horizontally inclined being in close proximity or in contact with the M2, and periodontal problems are found in 48% of M2s after extraction of the adjacent M3, with post-operative residual probing depth > 7 mm in 43.3% [1,6,7,8,9].

In order to prevent periodontal complications and assist the bone reconstruction of the sites after the surgical removal of M3s in such cases, and especially for patients older than 26 years, clinicians might need to implement additional measures, and guided bone regeneration techniques with the use of bone grafts are commonly considered for the reconstruction of the M3 extraction socket [1,3,10,11,12,13,14,15,16,17].

According to the available scientific evidence, different flap designs [13,14] or different suturing techniques [5] do not influence the periodontal healing of the M2, when removing M3s. On the contrary, systematic reviews have shown that bone grafting as a surgical intervention during removal of M3s may affect the outcome. For this reason, it is of great clinical importance to further document and evaluate the effect of the use of bone grafting materials and techniques when surgically extracting M3s with periodontal pathology on the distal aspect of the M2s.

The biomaterials that are used for bone grafting have different properties regarding new bone formation and graft resorption, mainly depended on their origin and chemical composition, thus leading to different amounts and quality of regenerated bone at the extraction site where they are implanted [18,19,20,21,22,23,24]. Randomized controlled trials analyzing and comparing data from human bone biopsies [25] have revealed that sockets treated with alloplastic biomaterials had the highest amount of regenerated bone (45.53%) compared to sites subjected to spontaneous healing with no graft material (41.07%) or xenografts (35.72%). The same studies have shown that the amount of residual biomaterial was highest in healed extraction sites grafted with xenografts (19.3%) compared to alloplastic materials (13.67%).

Although the body of literature pertaining the bone regeneration techniques aimed at preserving the periodontal health of the M2 after surgical removal of the adjacent M3 is not scarce, there are no studies investigating the use of calcium phosphates or calcium sulfate in such clinical scenarios.

Bioactive ceramics, such as β-TCP, are biocompatible grafts frequently utilized in bone regenerative procedures. As they are not from human or animal origin, they do not carry any immunological or infection risk. Their composition is similar to that of natural bone and can integrate with the defect site. Calcium phosphates and β-TCP are osteoconductive because osteoblasts adhere to them and deposit new bone on their surface [24,25,26,27,28,29,30]. A growing body of literature in the medical and dental research fields reveals and demonstrates the osteoinductive potential of novel calcium phosphate materials and the up-regulation of host regeneration as a result. These biomaterials can induce bone regeneration in extraskeletal areas by stimulating stem cells to differentiate to bone forming cells [31,32,33,34,35]. Moreover, β-TCP may also promote the proliferation and differentiation of endothelial cells, and improve the neovascularization of the grafted area, enhancing the bone reconstruction and function [36].

Calcium Sulfate has been in medicine for more than a century. It is a safe, fully-resorbable, mouldable material that has good handling properties and has been shown to support bone regeneration [37,38]. Adding CS to β-TCP produces a compound alloplastic scaffold that hardens in situ, binds directly to the host bone and helps maintaining the space and shape of the grafted site. The improved mechanical stability of the graft is an important factor for bone healing and differentiation of mesenchymal cells to osteoblasts, thus promoting regeneration of high-quality bone, as demonstrated in animal and human studies [39,40,41,42,43,44,45,46,47,48]. Moreover, the in situ hardening CS element may act as a cell occlusive barrier membrane halting soft tissue proliferation into the graft during the initial stages of healing [37,41].

Both CS and β-TCP are soluble bone substitutes, being degradable and fully replaced by new bone. The CS element is completely dissolved within 3–6 weeks after implantation, thus increasing the porosity in the β-TCP scaffold for improved vascular ingrowth and angiogenesis, while the β-TCP element will degrade by hydrolysis and phagocytosis, so that it will be completely substituted by new bone within 9–18 months [29,38,49,50,51].

The aim of this case series was to present and evaluate the clinical and radiological outcomes in patients older than 26 years with mesioangular or horizontally impacted M3s and periodontal pathology on the distal surface of the M2, treated with surgical removal of the M3 and simultaneous grafting of the osseous defect using a resorbable alloplastic β-TCP/CS biomaterial.

## 2. Materials and Methods

This prospective case series included patients aged 26 years or older who presented with the need of M3 surgical removal, horizontal or mesioangular impaction of the M3, radiological absence of bone between the crown of the M3 and the distal aspect of the adjacent M2 and good general health. Participants were recruited and treated in a private clinic in London, UK, between June 2017 and February 2020. All surgical procedures were performed by the same surgeon (M.L.) who is an experienced clinician and researcher specialized in Dentoalveolar Surgery. The patients were informed about the treatment, and gave their written consent for the use of the data for publication. Standard exclusion criteria for oral surgery procedures, such as allergy, uncontrolled systemic diseases, alcoholism and drug abuse, pregnancy or lactation, were applied. Heavy smokers (≥11 cigarettes/day) were also excluded.

**Figure 1:**
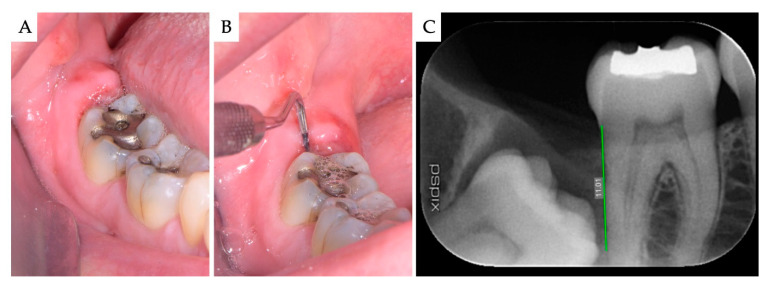
Case no 1. Impacted mesioangular M3 with pericoronitis. (**A**) Clinical view at presentation, showing the inflamed soft tissues distally to the M2; (**B**) PD was clinically recorded; and (**C**) the amount of BD was calculated on the digital periapical X-ray, using the measurement tool of the imaging software.

Radiographic examinations were made preoperatively, immediately postoperatively and at each follow up appointment. In every time point, standardized digital periapical X-rays were taken using phosphor plates fitted on commercially available holder devices (Kerr, Uxbridge, UK). A cone-bean computerized tomography (CBCT) was also taken before surgery in cases where further assessment was needed regarding the position and anatomy of the M3s, the extent of the pathology in the area, and the position of the inferior alveolar nerve. To evaluate the BG, the amount of BD was measured on the digital periapical X-rays utilizing image analysis software (Sopro Imaging Software, Acteon, UK) at the time of surgical removal of the M3 (T0) and at the final follow-up (T1). Follow-up was variable (minimum 1 year and maximum 2 years post-operatively). The amount of BD was defined as the distance between the cementoenamel junction of the second molar and the bottom of the bony defect on the distal surface of the M2 [1,11,12]. PD was clinically recorded at T0 and T1, measured with a periodontal probe at the central portion of the distal aspect of M2, as the distance from the free gingival margin to the bottom of the periodontal pocket (Figure 1). In cases of horizontal M3 impaction, the accurate initial PD measurement was not feasible, as the crown of the M3 was in direct contact with the distal aspect of the M2.

In cases of pericoronitis associated with the M3, broad spectrum antibiotics were prescribed for 5 days prior to surgery. Additionally, the sulcus was irrigated with oxygen-releasing solution (blue^®^m, Zwolle, The Netherlands) to dilute the bacterial population and remove any food trapped under the inflamed soft tissues.

The following procedure was planned for all cases. Under local anesthesia, a full-thickness buccal flap was raised. For fully impacted M3s a standard triangular (bayonet) flap was used, while when M3s were partially erupted a Szmyd flap design was preferred to facilitate advancement of the flap and primary closure. Bone was removed with a round burr under copious irrigation with sterile saline in order to adequately expose the crown of the M3. Subsequently, the tooth was sectioned into pieces which were mobilized and removed individually with the use of thin elevators and root luxators. All extraction sockets were thoroughly curetted to remove granulation tissue, followed by rinsing with sterile saline. Attention was given not to leave any remnants of soft tissues on the distal aspect of the root of the M2, which was scaled along with root planing.

**Figure 2:**
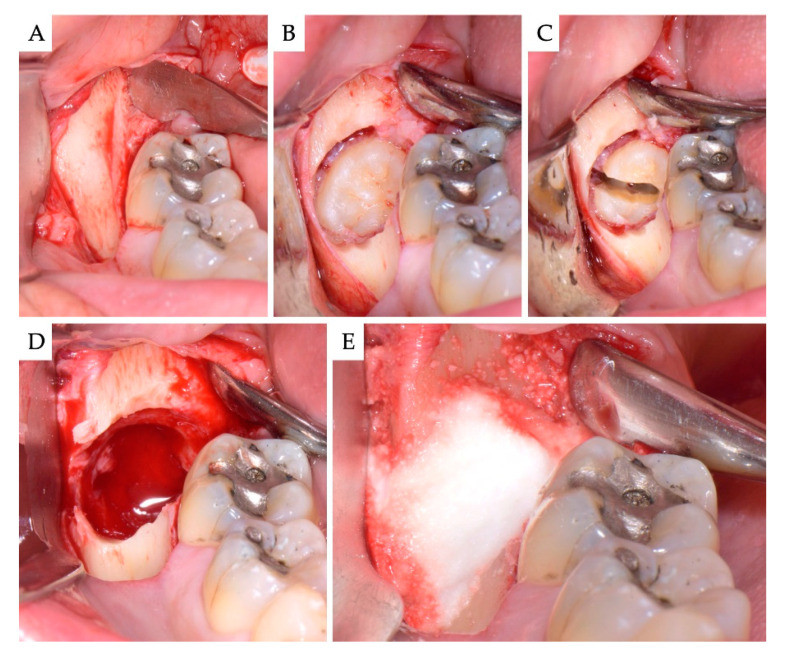
Case no 1. Impacted mesioangular M3. (**A**) Full-thickness standard triangular (bayonet) flap raised; (**B**) the crown of the M3 was exposed removing bone buccally, and (**C**) the tooth was sectioned and divided in half along its longitudinal axis. (**D**) The large post-extraction bone defect. (**E**) The site was grafted with the alloplastic β-TCP/CS (EthOss). No membranes were used.

**Figure 3:**
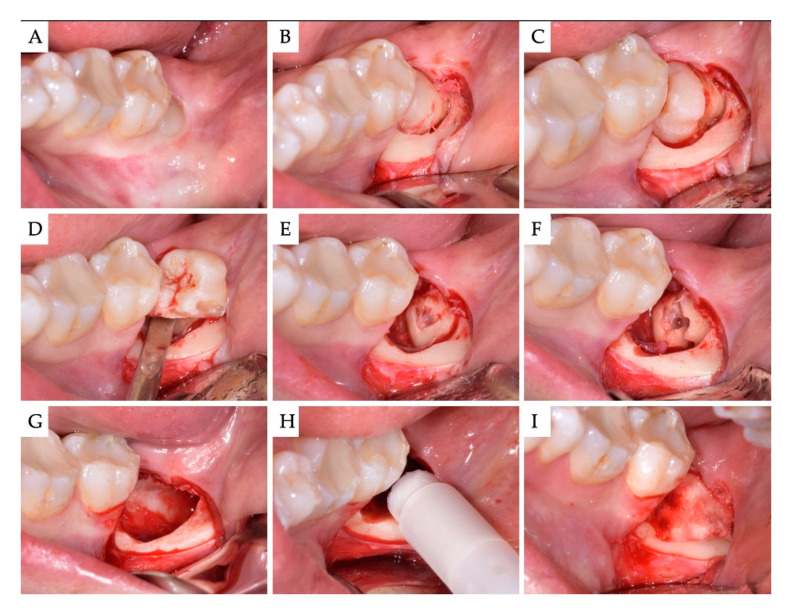
Case no 2. (**A**) Semi-impacted horizontal M3. (**B**) Full-thickness Szmyd flap raised. The crown of the M3 was adequately exposed by removing bone buccally. (**C**) The crown was separated from its roots and (**D**) luxated first. (**E**,**F**) The roots were sectioned and removed individually into the space vacated by the crown. (**G**) The bone defect after removal of the M3, curettage and rinsing with sterile saline. (**H**,**I**) Grafting with β-TCP/CS (EthOss). No membranes were used.

The bone defect was grafted using a self-hardening fully-resorbable alloplastic bone substitute (EthOss, Ethoss Regeneration Ltd., Silsden, UK), which consists of β-TCP (65%) and CS (35%). Prior to application the graft particles were mixed with sterile saline into the carrier syringe, and subsequently injected directly into the defect. The biomaterial was then gently condensed in situ using a bone plugger, while further compaction of the graft with a saline-wet gauze allowed for its setting, resulting to a stable scaffold for host osseous regeneration. No barrier membranes were used (Figure 2 and Figure 3). The flap was repositioned and sutured without tension with 5-0 monofilament sutures (SKD^®^ MONO, Miromed, Lainate, Italy), obtaining primary closure. Antibiotic therapy consisting of 500 mg amoxicillin every 8 h for 5 days and mouth rinsing with oxygen-releasing mouthwash (blue^®^m, Zwolle, Netherlands) every 8 h for 10 days were prescribed. The sutures were removed one week post-operatively.

## 3. Results

The final results are shown in Table 1.

In total, 4 patients (2 women and 2 men) with a mean age of 40.75 years (range: 34 to 51) were included in this prospective case series. All patients were non-smokers. Three M3s were horizontal (cases 2,3,4) and 1 had mesioangular impaction (case 1). In all cases, at presentation there was no bone radiologically on the distal aspect of the M2. In the cases of horizontal impactions, the crown of the M3 was in direct contact with the root surface of the M2, without any bone present in between. At T0 PD could be clinically measured only in case 1, where the crown of the impacted tooth was in distance from the distal surface of the M2, creating a clinically detectable 12 mm periodontal pocket. At baseline, the mean amount of BD was 9.33 ± 1.43 mm.

**Figure 4:**
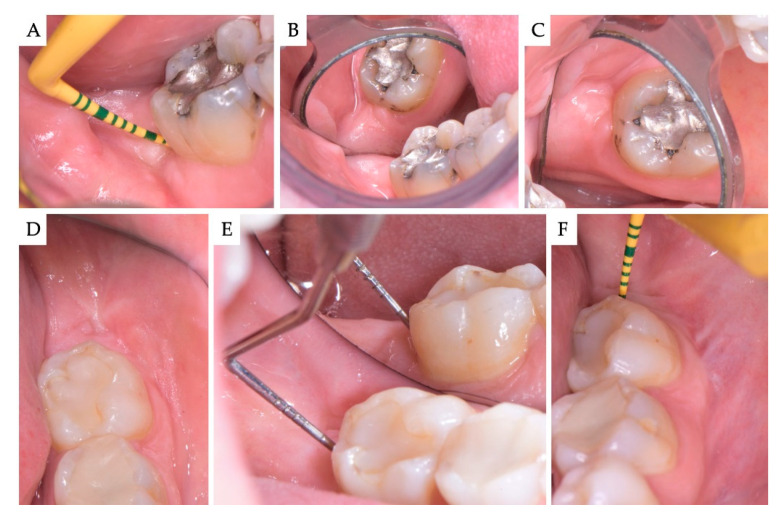
(**A**–**C**) Case no 1; Clinical views 2 years post-operatively. Healthy periodontal tissues with no bleeding on probing and 3 mm probing depth on the distal aspect of the M2. The site is covered with keratinized soft tissues, and the architecture of the ridge is adequately restored. (**D**,**E**) Case no 2; clinical view 6 months post-operatively and (**F**) 2 years post-operatively. No residual periodontal pockets on the distal aspect of the M2 at any time point.

**Figure 5:**
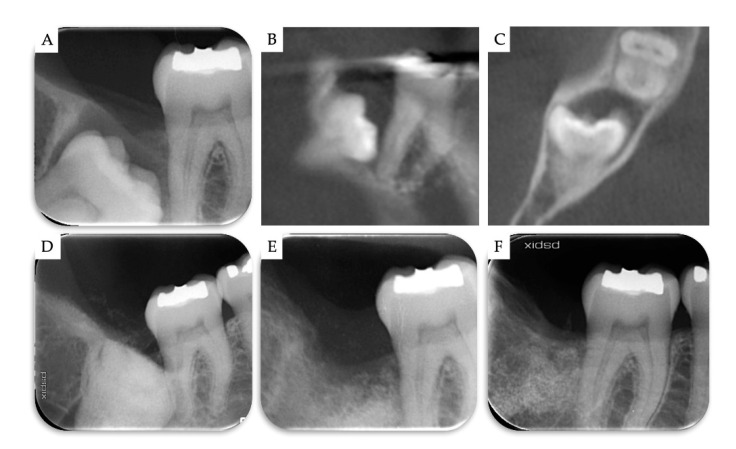
Case no 1. (**A**–**C**) Periapical X-ray and CBCT images at T0. Periapical X-rays (**D**) immediately after the removal of M3 and grafting with β-TCP/CS, (**E**) 4 months post-operatively, and (**F**) 2 years post-operatively.

**Figure 6:**
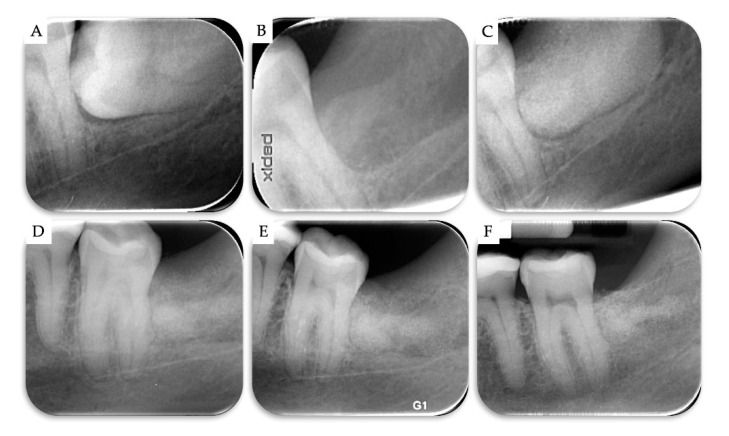
Case no 2. Periapical X-rays (**A**) at T0, (**B**) after the surgical removal of the horizontal M3, (**C**) immediately after grafting the extraction site with β-TCP/CS, (**D**) 6 months post-operatively, (**E**) 9 months post-operatively, and (**F**) 2 years post-operatively.

**Figure 7:**
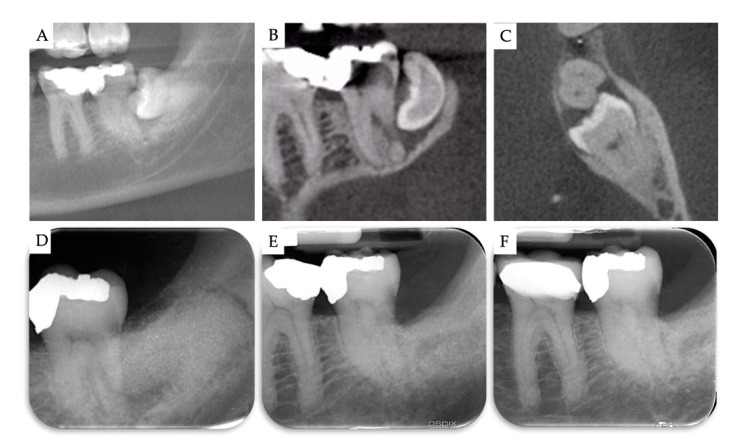
Case no 3. (**A**–**C**) CBCT images at T0. Periapical X-rays (**D**) immediately after the removal of M3 and grafting with β-TCP/CS, (**E**) 10 months post-operatively, and (**F**) 1 year post-operatively.

**Figure 8:**
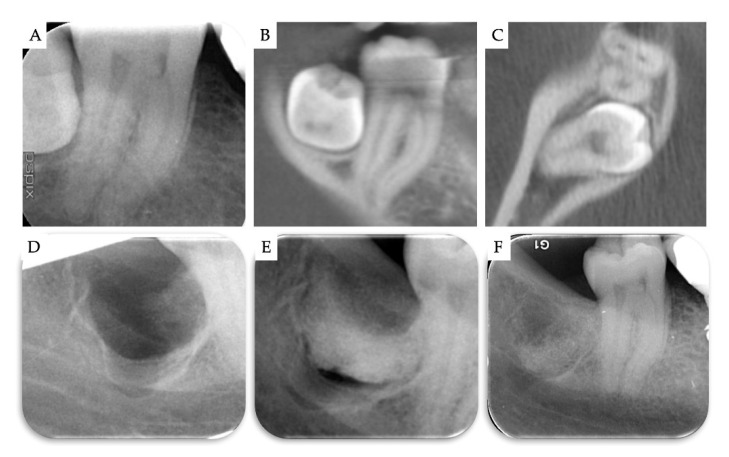
Case no 4. (**A**–**C**) Periapical X-ray and CBCT images at T0. Periapical X-ray (**D**) immediately after the removal of M3, and (**E**) immediately after grafting the bone defect with β-TCP/CS. (**F**) 1 year post-operatively.

No intra- or post-operative complications occurred. The healing in all cases was uneventful, without wound dehiscence, nor loss of grafting material. The mean follow-up period was 1.5 year (range 1 to 2 years). Radiological follow-up examination with periapical X-rays showed the successful incorporation of the grafting material and the regeneration of new bone in all grafted sites, in parallel to the gradual turnover of the resorbable β-TCP/CS biomaterial. At the endpoint of this case series, PD was 2.00 ± 0.71 mm, with no periodontal pathology on the distal aspect of the M2. The amount of BD at T1 was 3.25 ± 1.6 mm, corresponding to a BG of 6.07 ± 0.28 mm (Figure 4, Figure 5, Figure 6, Figure 7 and Figure 8).

No post-operative CBCTs were taken. As in all cases, clinical examination and periapical X-rays showed successful outcomes with no residual pathology on the distal aspect of the M2, and as no further treatment or intervention was planned in this area, there was no justification to perform additional radiological assessment with follow-up CBCTs.

## 4. Discussion

The relatively high prevalence of having a periodontal defect on the distal aspect of M2 molar after Μ3 extraction makes it necessary to develop the appropriate treatment modalities in order to prevent such complications. It has been shown that more than 40% of M2s had an intrabony defect of at least 4 mm, and more than 50% of M2s had a PD of at least 7 mm even 4 years after third-molar extraction [52].

The results of the present prospective case series indicate that the use of an alloplastic grafting material, consisting of β-TCP and CS, resulted in significant bone gain and effectively prevented periodontal defect on the distal aspect of M2, when used for treating osseous defects after the surgical removal of mesioangular or horizontal M3s in patients older than 26 years. Our results are in accordance with the findings of recent systematic reviews and meta-analyses showing that regenerative periodontal therapy is effective in preventing the distal periodontal defect of M2 after M3 extraction with regard to clinical attachment level gain, PD reduction, and BG, without increasing the risk of postoperative complications [2,3].

In this case series, the use of β-TCP/CS resulted in significant BG of 6.07 ± 0.28 mm, while PD distally to the M2 was 2.00 ± 0.71 after a follow-up period of 1.5 ± 0.5 years. Only one randomized clinical trial evaluating the use of bone grafting has revealed similar results in BG. In this trial, Ge et al. (2017) investigated the effect of autogenous bone grafting compared with non-grafting after impacted M3 removal. After 12 months, BG of 5.6 ± 2.5 mm was observed at the sites treated with autogenous bone, compared to 3.6 ± 1.4 mm at sites where spontaneous healing was allowed. PD for the grafted sites was 3.0 ± 1.0 mm and 3.5 ± 1.4 for the non-grafted sites. The authors reported that the need for harvesting the autogenous bone resulted in longer operative times, while these patients experienced more swelling and pain, compared to patients that did not receive autogenous grafting [12]. Inferior results were reported by Hassan et al. (2012) evaluating the use of bovine xenograft plus a resorbable barrier membrane for periodontal osseous defects distal to the M2 compared with non-grafted extraction sites after removal of impacted M3s in patients 30- to 35-years-old. Grafting of osseous defects distally to M2s with an anorganic xenograft plus a membrane predictably resulted in a BG of 3.59 ± 1.14 mm 12 months post-operatively, compared to 1.20 ± 1.32 mm for the non-grafted sites. PD for the grafted sites was 3.1 ± 0.4 mm and 4.9 ± 0.5 mm for the non-grafted sites [17]. Dentin autogenous graft can be used to graft the bone defect after removal of M3s, as an alternative to autogenous bone or other bone substitutes. De Biase et al. (2020) reported a split-mouth case where an 18-year-old patient underwent surgery of both impacted M3s [53]. The right extraction socket was grafted with autologous dentin graft (test side), while the left site was filled with fibrin sponge (control). Six months post-operatively, the test site, treated with grinded dentin, was characterized by a minor depth of the pocket compared with the nongrafted site, with no clinical/radiographic signs of complications. Radiographic measurements using periapical X-rays revealed significant BG (2.612 mm before the surgery and 0.287 mm 6 months post-operatively) for the grafted site, compared to the control site (1.598 mm and 0.658 mm, respectively).

Although barrier membranes seem to be more effective than bone grafting in such clinical scenarios [2,3], the use of resorbable or non-resorbable membranes has several drawbacks. Non-resorbable titanium reinforced membranes are associated with soft-tissue complications, as wound dehiscence and exposure of the membrane to the oral cavity will require early membrane removal, resulting in impaired periodontal regeneration. An additional disadvantage of non-resorbable membranes is the need for a second surgical procedure for membrane removal, resulting in higher patient morbidity and prolonged overall treatment time. Resorbable membranes have been proposed to overcome these disadvantages. However, early degradation, epithelial downgrowth along the material and premature loss of material have been reported following the use of resorbable membranes [2,54,55]. In the present case series, no membranes were used as the CS element of the biomaterial used helped stabilize the graft and provided a barrier function. This led to simplified surgical procedures and less cost, while allowing the alloplastic graft to be in direct contact to the periosteum which possesses an immense osteogenetic potential and its role on bone regeneration should not be under-estimated [26,55,56].

It is important that even with promising results of BG and PD reduction with the use of bone grafting materials reported in the literature, it is difficult to conclude that regeneration of periodontal tissues happens on the distal site of M2s without seeing histologic results [2]. As recent studies in bone reconstruction are gradually shifting their focus onto biodegradable and bioactive materials, clinicians should aim in regenerating high-quality new bone, without the long-term presence of non-resorbing graft particles, which might act as foreign materials. In this case series, a bioactive fully-resorbable alloplastic material was used in an attempt to enhance the reconstruction of high quality bone in the osseous defects, for complete regeneration up to the condition of restitutio ad integrum, and not to serve just as a filling material. The β-TCP/CS bone graft used in the presented cases has been researched in preclinical and clinical studies, showing that such biomaterials can accelerate and enhance the regeneration of high quality host vital bone, without the need for the use of additional barrier membranes [43,44,45,46,47,48].

## 5. Conclusions

This paper reports that in a series of patients older than 26 years with mesioangular or horizontally impacted M3s and periodontal pathology on the distal surface of the M2, surgical removal of the M3 and immediate grafting of the osseous defect with β-TCP/CS resulted in successful healing and stable outcomes after a follow-up period of 1 to 2 years. Within the limitations of the present prospective case series (small sample size, no comparative group), the use of the β-TCP/CS bone substitute in these cases seemed to be effective in reconstructing the bone locally, thus preventing post-operative periodontal complications on the distal aspect of the M2. Further research, including larger samples and comparison of different materials and methods, is needed in order to confirm and supplement the present findings.

## Figures and Tables

**Table 1:** Patients’ characteristics; follow-up period; pocket depth (PD), bone defect (BD) and bone gain (BG) measurements.

Case	Gender	Age	Smoker	M3	Impaction	Follow-Up (years)	PD T0 (mm)	PD T1 (mm)	BD T0 (mm)	BD T1 (mm)	BG (mm)
1	F	51	No	48	Mesio-angular	2	12	3	11.1	5.2	5.86
2	F	36	No	38	Horizontal	2	-	1	7.4	1.3	6.1
3	M	42	No	38	Horizontal	1	-	2	10.2	4.4	5.79
4	M	34	No	48	Horizontal	1	-	2	8.6	2.1	6.51
Mean		40.75				1.5		2.00	9.33	3.25	6.07
SD		6.61				0.5		0.71	1.43	1.6	0.28
